# Identification of Two Critically Deleted Regions within Chromosome Segment 7q35-q36 in *EVI1* Deregulated Myeloid Leukemia Cell Lines

**DOI:** 10.1371/journal.pone.0008676

**Published:** 2010-01-13

**Authors:** An De Weer, Bruce Poppe, Sarah Vergult, Pieter Van Vlierberghe, Marjan Petrick, Robrecht De Bock, Yves Benoit, Lucien Noens, Anne De Paepe, Nadine Van Roy, Björn Menten, Frank Speleman

**Affiliations:** 1 Centre for Medical Genetics, Ghent University Hospital, Ghent, Belgium; 2 Department of Radiotherapy, Oncology and Hematology, AZ Sint-Lucas, Ghent, Belgium; 3 Department of Hematology, ZNA Middelheim, Antwerp, Belgium; 4 Department of Pediatric Hemato-Oncology, Ghent University Hospital, Ghent, Belgium; 5 Department of Hematology, Ghent University Hospital, Ghent, Belgium; Innsbruck Medical University, Austria

## Abstract

Chromosomal rearrangements involving the *EVI1* proto-oncogene are a recurrent finding in myeloid leukemias and are indicative of a poor prognosis. Rearrangements of the *EVI1* locus are often associated with monosomy 7 or cytogenetic detectable deletions of part of 7q. As *EVI1* overexpression alone is not sufficient to induce leukemia, loss of a 7q tumour suppressor gene might be a required cooperating event. To test this hypothesis, we performed high-resolution array comparative genomic hybridization analysis of twelve *EVI1* overexpressing patients and three *EVI1* deregulated cell lines to search for 7q submicroscopic deletions. This analysis lead to the delineation of two critical regions, one of 0.39 Mb on 7q35 containing the *CNTNAP2* gene and one of 1.33 Mb on chromosome bands 7q35–q36 comprising nine genes in *EVI1* deregulated cell lines. These findings open the way to further studies aimed at identifying the culprit *EVI1* implicated tumour suppressor genes on 7q.

## Introduction

Chromosomal rearrangements involving chromosome band 3q26, such as translocations with various partner chromosomes or inversions of chromosome 3 are a recurrent finding in myeloid leukemias [1]. These aberrations contribute to the ectopic expression of the *EVI1* proto-oncogene. *EVI1* transcriptional activation has been reported in up to 10% of myeloid leukemia patients, even in the absence of 3q26 rearrangements, and is an independent indicator of adverse prognosis [2].

Retroviral integration experiments have shown that *EVI1* overexpression alone is not sufficient to cause leukemia, indicating that cooperative effects are necessary for malignant transformation [3], [4]. Interestingly, over fifty percent of the 3q26 rearranged leukemias also display chromosomal abnormalities involving chromosome 7, such as monosomy 7 or deletion of part of 7q [5]. This association alludes to the existence of a 7q tumour suppressor gene, which when deleted acts in concert with *EVI1* overexpression to induce malignant transformation. We performed high-resolution array comparative genomic hybridization (CGH) analysis to search for 7q submicroscopic deletions in *EVI1* deregulated leukemia patients in order to identify candidate 7q tumour suppressor genes.

## Materials and Methods

### Patients and Cell Lines

Diagnostic bone marrow samples of twelve myeloid leukemia samples and remission bone marrow samples of two patients (case 7 and case 8) were included in the study. Fluorescence *in situ* hybridization (FISH) for detection of *EVI1* rearrangement and reverse transcription quantitative PCR (RT-qPCR) for detection of *EVI1* ectopic expression was performed as previously described [6]. The study was approved by the ethics committee of the Ghent University Hospital (2003/273).

Three *EVI1* rearranged cell lines, Kasumi-3, MUTZ-3 and UCSD-AML1 were also included in the study [7], [8]. For the cell lines culture conditions were as follows, for Kasumi-3 RPMI-1640 medium (Invitrogen, Belgium) was supplemented with 15% foetal calf serum, 1% penicillin/streptomycin, 1% kanamycin, 1% glutamine, 2% HEPES (1 M), 1% sodiumpyruvate (100 nM) and 0.1% beta-mercapto ethanol (50 nM). For MUTZ-3, the used medium contained an extra 10% of the supernatant of the 5637 urinary bladder carcinoma cell line [9] and 10 ng/ml of GM-CSF (Promocell, UK). For the UCSD-AML1 cell line, the used medium contained an additional 10 ng/ml of GM-CSF (Promocell, UK). Patient and cell line characteristics and *EVI1* FISH and RT-qPCR results are described in Table 1.

**Table 1:** Patient and cell line characteristics: diagnosis, karyotype and *EVI1* FISH and RT-qPCR results.

Name	Diagnosis*	Karyotype†	FISH *EVI1* ‡	RT-qPCR *EVI1* ‡
Kasumi-3	AML	46,XY,t(2;5)(p13;q33),**t(3;7)(q26;q22)**,del(5)(q15),−8,del(9)(q32),add(12)(p11),add(16)(q13),+mar[20]	+	+
MUTZ-3	AML	46,XY,t(1;3)(q43;q13),t(2;7)(q36;q36),**inv(3)(q21q26)**,inv(7)(p15q36),t(12;22)(p13;q12)[20]	+	+
UCSD-AML1	AML	45,XX,t(2;22)(p13;q12),**t(3;3)(q21;q26)**,−7[20]	+	+
case 1	MDS	46,XX,**t(3;8)(q26;q23)** [13]/46,XX[1]	+	+
case 2	AML	47,XY,+8,**inv(3)(q21q26)**,t(13;22)(q10;p10)[20]	+	+
case 3	AML	46,XY,**t(3;21)(q26;q22)**,add(17)(p11.2)[12]/46,XY[5]	+	+
case 4	MDS	45,XY,add(1)(q13),**t(3;21)(q26;q22)**,add(5)(q13),−13,−18,+22[10]/46,XY[6]	+	+
case 5	AML	46,XY,**inv(3)(q21q26)** [8]/46,XY[2]	+	+
case 6	AML	47,XX,t(9;11)(p22;q23),del(11)(p15),+21[8]/46,XX[12]	−	+
case 7	AML	46,XX,t(9;11)(p22;q23)[8]/46,XX[12]	−	+
case 8	AML	45,X,−X,t(9;11)(p22;q23)[12]/46,XX[6]/45,X, −X[3]	−	+
case 9	AML	45,XY,**inv(3)(q21q26)**, −7[17]	+	+
case 10	AML	46,XY,**t(3;3)(q21;q26)** [15]	+	+
case 11	AML	45,XX,**add(3)(q26)**, −7[15]	+	+
case 12	MDS	45,XY,**t(3;21)(q26;q22)**, −7[20]	+	+

Bold formatting indicates the 3q26 rearrangement.

* AML = acute myeloid leukemia and MDS = myelodysplastic syndrome.

† The chromosomal aberration implicating the *EVI1* locus is indicated with bold formatting.

‡ Positive for *EVI1* FISH/qRT-PCR = +and negative for *EVI1* FISH/qRT-PCR = −.

### Array CGH

DNA of patient samples and cell lines was isolated using the QIAamp DNA mini kit (Qiagen, Belgium) or the Puregene Cell kit (Gentra Systems, Belgium) according to the manufacturer's descriptions. Array CGH analysis for submicroscopic 7q deletions was performed on a 44K custom array (Agilent technologies, Belgium) covering the entire chromosome 7 with a probe spacing of 1 oligonucleotide every 3 kb, according to the manufacturer's descriptions. In brief, DNA (400 ng) was labelled using the BioPrime Array CGH genomic labelling system (Invitrogen, Belgium) using Cy5 (control DNA; Promega, Belgium) and Cy3 (patient sample or cell line) labelled dCTPs (GE Healthcare, Belgium). Following labelling, hybridization, and washing of the slides, arrays were scanned using an Agilent DNA Microarray Scanner, quantified with Feature Extraction software 10.1 and data were further analyzed with arrayCGHbase [10] using a circular binary segmentation (CBS) algorithm taken into consideration log_2_ ratios of neighbouring probes [11]. Deletions were called heterozygous when CBS ratios were ≤−0.5 and a deletion was considered putatively homozygous when the CBS ratios were ≤−1.2. Common copy number variations (CNVs) present in the Database of Genomic Variants (www.projects.tcag.ca/variation) were excluded from analysis.

### Fluorescence In Situ Hybridization

To confirm the overlapping deletions found in the cell lines Kasumi-3, UCSD-AML1 and MUTZ-3 at 7q35-q36, FISH analysis was performed as previously described [6]. FISH probes were selected from the UCSC genome browser database (http://genome.ucsc.edu) (Table 2).

**Table 2:** Name and position of FISH probes.

Probe name	Position start*	Position end*
RP11−114L10	142486383	142604687
RP11−106C6	148240833	148415979
RP11−171N15	141216856	141779639
RP11−728K20	149582837	149731647
RP11−418B24	146512768	146667113
RP11−1123J16	146673416	146811717
RP4−803F10	146336163	146439692
RP4−777G9	146949516	147038612
RP11−79O14	147671295	147818484
RP11−452J20	151938144	152144780
RP11−312C1	152459779	152639208

* UCSC genome browser database (http://genome.ucsc.edu).

### CNTNAP2 Expression Analysis

Total RNA was extracted from total bone marrow samples of thirty nine *EVI1* overexpressing patients (nine with monosomy 7), five normal bone marrow samples and two CD34^+^ cell fractions, using the miRNeasy kit (Qiagen, Belgium). cDNA was prepared from 2 µg of total RNA with the iScript cDNA Synthesis Kit (Bio-Rad, Belgium) according to the manufacturer's descriptions and RT-qPCR for the *CNTNAP2* (forward: 5′-TAGGACATGGAACCCCAATG-3′ and reverse: 5′-ATCGATTTGGCTCATCTTGG-3′) transcript was performed as previously described [12], [13]. The reference genes *RPL13A* (forward: 5′-CCTGGAGGAGAAGAGGAAAGAGA-3′ and reverse: 5′-TTGAGGACCTCTGTGTATTTGTCAA-3′) and *YWHAZ* (forward: 5′-ACTTTTGGTACATTGTGGCTTCAA-3′ and reverse: 5′-CCGCCAGGACAAACCAGTAT-3′) were used for normalisation of the RT-qPCR data.

## Results and Discussion

**Figure 1:**
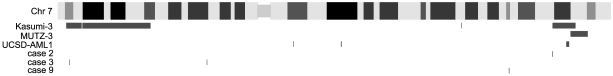
Overview of deletions in cell lines and patients. Chromosome view of chromosome 7 deletions in the cell lines Kasumi-3, MUTZ-3 and UCSD-AML1 and in cases 2, 3 and 9. Deletions are indicated using grey bars.

**Figure 2:**
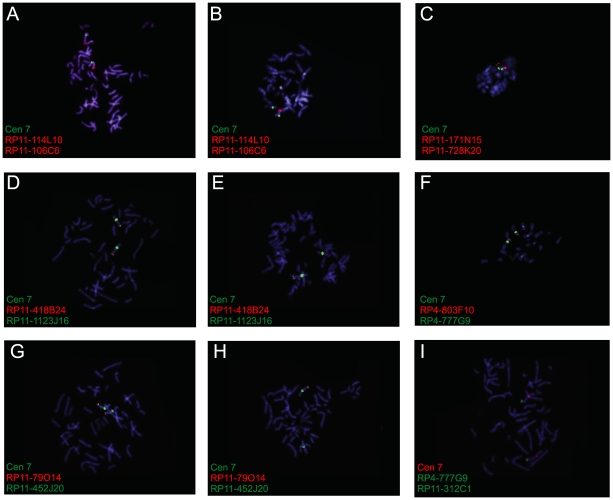
FISH analysis of the 7q35–q36 deletions in cell lines. A), D) and G) FISH analysis on cytogenetically normal controls with probes located within the 7q35–q36 deleted regions. B), E) and H) FISH analysis with probes located within the 7q35–q36 deleted regions on the cell lines Kasumi-3 (B), UCSD-AML1 (E) and MUTZ-3 (H). C), F) and I) FISH analysis with probes just outside of the 7q35–q36 deleted regions on the cell lines Kasumi-3 (C), UCSD-AML1 (F) and MUTZ-3 (I).

Based on karyotyping and FISH analysis, two critical regions, one on chromosome band 7q22 [14] and one on chromosome bands 7q32–q35 [15], have been identified as the targets for 7q deletions in myeloid leukemia. In this study, array CGH was applied to search for deletions on chromosome 7 at ultra-high resolution. Using this approach, we identified several submicroscopic 7p and 7q deletions in *EVI1* overexpressing patients and cell lines (Table 3 and Figure 1) including two critically deleted regions on 7q35–q36 in *EVI1* deregulated cell lines. These deletions were confirmed using FISH (Figure 2).

**Table 3:** Array CGH results of chromosome 7 deletions.

Name	Chromosomal position*	Start - end (Mb)	Size (Mb)	Genes†
Kasumi-3	7p22	2369956−6743766	4,37	*SDK1* (40)
	7p22−p15	7010834−26480795	19,47	63
	**7q31.2**	**116412304**−**116584949**	**0,17**	***ST7***
	**7q34**−**q36**	**142182589**−**148693649**	**6,51**	***KEL, CNTNAP2*** ‡ **, ** ***CUL1*** **, ** ***EZH2 (48)***
MUTZ-3	**7q35**−**q36**	**147377325**−**152205764**	**4,83**	***CNTNAP2, CUL1, EZH2 (61)***
UCSD-AML1	**7q11.22**	**69266162**−**69318942**	**0.053**	***AUTS2*** ‡
	**7q21.11**	**78183200**−**78189877**	**0.007**	***MAGI2*** ‡
	**7q35**	**146482743**−**146873269**	**0.391**	***CNTNAP2*** ‡
case 2	**7q34**	**142351441**−**142361904**	**0,01**	***KEL***
case 3	7p22	4036389−4042650	0,01	*SDK1*
	7p12	43592619−43597672	0,01	*STK17A*
case 9	**7q32.2**	**129076051**–**129078116**	**0.02**	***NRF1*** ‡

Bold formatting indicates deletions on the 7q arm.

* Based upon the UCSC genome browser (http://genome.ucsc.edu/cgi-bin/hgGateway, NCBI Build 36.1).

† Between brackets is the total number of genes residing in the deleted area.

‡ Homozygous deletion.

**Figure 3:**
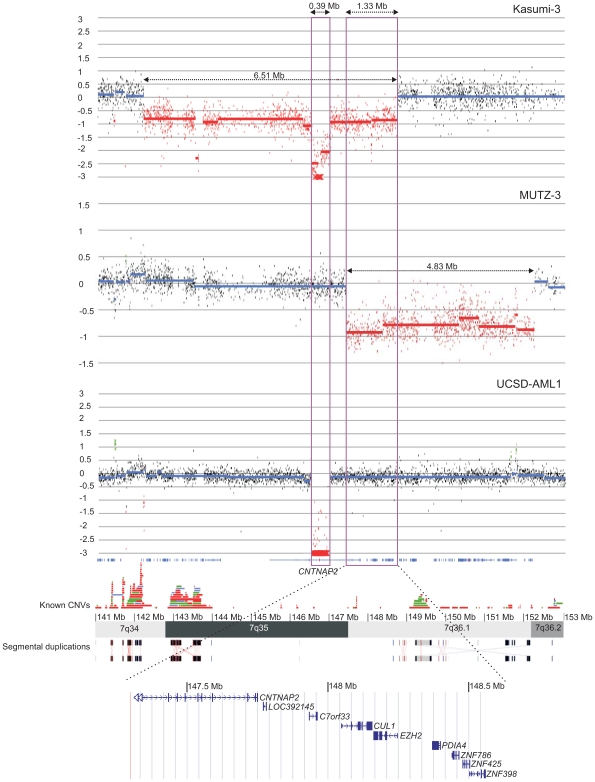
Array CGH profile of chromosome 7 (141 Mb–153 Mb) for Kasumi-3, MUTZ-3 and UCSD-AML1. A) Array CGH profile of Kasumi-3, B) MUTZ-3 and C) UCSD-AML1 indicating the 0.39 Mb SRO on 7q35 and the 1.33 Mb SRO on chromosome bands 7q35–q36. Log_2_-ratios of the clones are depicted by vertical dots corresponding to the respective genomic position (NCBI build 36). Deletions are shown in red. The SROs are indicated with a purple box. The bottom of the figure shows the genomic position with an indication of known CNVs as present in the Database of Genomic Variants (http://projects.tcag.ca/variation/). Regions of segmental duplication are displayed using black and grey boxes indicating identical genomic regions. A screenshot of the UCSC Genome Browser (NCBI build 36, http://genome.ucsc.edu) shows an overview of the known RefSeq genes in the 1.33 Mb critical region.

Interestingly, in the Kasumi-3 cell line a 680 kb homozygous deletion on 7q encompassing the *CNTNAP2* gene was identified, located within a 6.51 Mb heterozygous deleted region at 7q34–q36. The observation of homozygous deletions has been of great importance in tumour genetics as the deleted region often contains putative tumour suppressor genes. In the MUTZ-3 cell line, a 4.83 Mb deletion was present within the same chromosomal region, delineating a shortest region of overlap (SRO) of 1.33 Mb (Figure 3) encompassing nine genes (Table 4). Interestingly, in the UCSD-AML1 cell line a homozygous deletion of 0.39 Mb was detected encompassing the *CNTNAP2* gene, which delineated a second SRO of 0.39 Mb on 7q35 (Figure 3).

**Table 4:** Genes located in the 1.33 Mb SRO on 7q35−q36.

Gene name	Annotation
*CNTNAP2*	contactin associated protein-like 2 isoform b
*LOC392145*	hypothetical protein LOC392145
*C7orf33*	hypothetical protein LOC202865
*CUL1*	cullin 1
*EZH2*	enhancer of zeste homolog 2
*PDIA4*	protein disulfide isomerase associated 4
*ZNF786*	zinc finger protein 786
*ZNF425*	zinc finger protein 425
*ZNF398*	zinc finger 398 isoform a

*CNTNAP2* is located in a common fragile site which is inactivated in different types of cancers such as brain, ovarian and breast tumours [16], and methylation of the promoter of this gene has been described in pancreatic adenocarcinoma [17]. However, upon RT-qPCR analysis of the *CNTNAP2* gene no expression could be detected in normal bone marrow samples, CD34^+^ cell fractions or *EVI1* overexpressing bone marrow samples (data not shown), indicating that this gene is not a straightforward candidate 7q tumour suppressor gene cooperating with *EVI1* overexpression.

Subsequent screening of twelve *EVI1* overexpressing patient samples with (3 cases) or without (9 cases) monosomy 7, revealed no submicroscopic alterations within the above described SROs. However, focal deletions in other genomic regions on chromosome arms 7p and 7q were detected.

Of interest is that both the Kasumi-3 and the MUTZ-3 cell line display chromosomal rearrangements involving chromosome 7. It is well known that small deletions can occur at chromosomal breakpoints [18]. Therefore, the observed 4.83 Mb deletion at 7q36 in MUTZ-3 might have originated as a consequence of the t(2;7)(q36;q36) and/or the inv(7)(p15q36) present in this cell line.

Of the nine genes located within the 1.33 Mb SRO *CUL1* and *EZH2* are the most promising candidates due to known function in and association with cancer. The *CUL1* gene encodes a protein that, when associated with other proteins such as Skp1, forms the Skp2 E3 ubiquitin ligase E3 complex involved in degradation of different proteins [19]. In AML, the Skp2 complex has been described to target the MEF transcription factor which induces G1/S transition. Impairment of the Skp2 complex leads to a decrease in MEF degradation thereby promoting cell proliferation [20]. Deletion and mutation of other E3 ubiquitin ligase complex members such as the F-box containing *FBXW7* gene has already been described in T-cell acute lymphoblastic leukemia (T-ALL) where it is associated with poor prognosis [21]. Moreover, in AML loss-of-heterozygosity (LOH) of the *EZH2* (polycomb group) gene (enhancer of zest) was detected in 5 out of 21 patients [22]. However, further analyses such as mutation screening and functional RNA interference screens [23] are needed to identify the genes contributing to *EVI1* leukemogenesis.

In addition to the regions described above, other 7q deletions in the *EVI1* overexpressing patients as well as in the UCSD-AML1 cell line were detected. In UCSD-AML1 two additional small 7q deletions were observed, a 53 kb (7q11.22) and 7 kb (7q21.11) deletion and containing the *AUTS2* and *MAGI2* genes respectively. In case 2, a small deletion of 10 kb on 7q34 was observed encompassing the *KEL* blood group gene. In Kasumi-3, this gene was located in a larger deleted region of 6.51 Mb on 7q34–q36. In case 9, a small homozygous deletion of 20 kb on 7q32.2 was detected. Located in this region is the *NRF1* gene for which inactivating mutations have already been described in liver cancer [24].

Besides the above mentioned 7q deletions, several 7p deletions were also detected, some of which were common between different patients and/or cell lines. In case 3, a small deletion of 10 kb on 7p22 was observed encompassing the *SDK1* sphingosine dependent protein kinase gene. In Kasumi-3, this gene was located in a larger deleted region of 4.37 Mb on 7p22. As involvement of the *SDK1* gene in apoptosis has already been described [25], deletion of this gene could lead to a decreased rate of cell death. For patient 3 deletion of the *STK17A* gene at 7p12 was detected. Deletion of this pro-apoptotic gene had already been described in laryngeal squamous cell carcinoma [26]. It should be noted that it is possible that the above described small 7q and 7p deletions in patient samples are not leukemogenic as no constitutional material of these patients was available. In the remaining patient samples no chromosome 7 deletions could be detected.

In conclusion, using high-resolution array CGH, we were able to delineate two critical regions for 7q deletions in myeloid leukemias that were not detectable using standard karyotyping. Besides a 0.39 Mb SRO on 7q35 containing the *CNTNAP2* gene, we identified a 1.33 Mb region on chromosome bands 7q35–q36 containing nine putative tumour suppressor genes in *EVI1* deregulated cell lines. Further analysis of these candidates is warranted to investigate their role in *EVI1* mediated malignant transformation. Identification of an *EVI1* cooperating 7q tumour suppressor gene opens perspectives for novel treatment strategies selectively restoring the expression of the deleted genes.
